# Gonadal Failure Is Common in Long-Term Survivors of Childhood High-Risk Neuroblastoma Treated With High-Dose Chemotherapy and Autologous Stem Cell Rescue

**DOI:** 10.3389/fendo.2019.00555

**Published:** 2019-08-08

**Authors:** Pauliina Utriainen, Anu Suominen, Outi Mäkitie, Kirsi Jahnukainen

**Affiliations:** ^1^Pediatric Research Center, Children's Hospital, Helsinki University Hospital, Helsinki, Finland; ^2^Research Program for Clinical and Molecular Metabolism, Faculty of Medicine, University of Helsinki, Helsinki, Finland; ^3^Folkhälsan Research Center, Helsinki, Finland; ^4^Department of Molecular Medicine and Surgery, Karolinska Institutet and Clinical Genetics, Karolinska University Hospital, Stockholm, Sweden; ^5^Nordfertil Research Laboratory Stockholm, Department of Women's and Children's Health, Karolinska Institute and University Hospital, Stockholm, Sweden

**Keywords:** high-risk neuroblastoma, irradiation, chemotherapy, late effect of cancer treatment, gonadal function, gonadal failure, fertility, puberty

## Abstract

**Background:** Neuroblastoma is the most common extra-cranial solid tumor in children. Intensive therapy including autologous stem-cell transplantation (HSCT) has improved the poor prognosis of high-risk neuroblastoma (HR-NBL) but may impair gonadal function.

**Objectives:** To investigate the gonadal function and fertility in long-term survivors of childhood HR-NBL.

**Design:** A cohort including all Finnish (*n* = 20; 11 females) long-term (>10 years) survivors of HR-NBL and an age- and sex-matched control group (*n* = 20) was examined at a median age of 22 (16–30) years. Oncologic treatments, pubertal timing, hormonal therapies and the number of off-spring were recorded, and pituitary and gonadal hormones were measured.

**Results:** Altogether 16/20 of the long-term survivors of HR-NBL entered puberty spontaneously; puberty was hormonally induced in four survivors (three females). Among the 8/11 female survivors with spontaneous puberty, seven had spontaneous menarche, but 5/8 developed ovarian failure soon after puberty. Nine females currently needed estrogen substitution. AMH, a marker of ovarian reserve, was lower in the female survivors than controls (median 0.02 vs. 1.7 μg/l, *p* < 0.001). As a group, male survivors had smaller testicular size (8.5 vs. 39 ml, *p* < 0.001) and lower inhibin B (<10 vs. 170 ng/l, *p* < 0.001) compared with control males, with altogether 6/9 survivor males fulfilling the criteria of gonadal failure (absent puberty, small testicle size or increased FSH with need of testosterone substitution). Gonadal failure was more common in female and male survivors treated with total-body irradiation. Three survivors (one male) had offspring, all treated without total-body irradiation and moderate dose of alkylating chemotherapy. Growth velocity was compromised in all survivors after HR-NBL diagnosis, with absent pubertal growth spurt in 7/17 survivors with complete growth data.

**Conclusion:** Gonadal failure is common in long-term survivors of HR-NBL treated with HSCT. Fertility may be preserved in some survivors treated without total-body irradiation.

## Introduction

Neuroblastoma (NBL) is the most common extra cranial malignant solid tumor in childhood. Approximately one-half of the NBL patients present with high-risk features (1, 2). The prognosis of high-risk neuroblastoma (HR-NBL) has long remained poor but improved with intensive multimodal therapy (1) including multi-drug induction chemotherapy, surgical resection of primary tumor with/without local irradiation, myeloablative therapy together with autologous hematopoietic stem-cell transplantation (HSCT), and 13-cis-retinoic acid consolidation (3–6).

Neuroblastoma is typically a disease of small children. Aggressive treatment at young age predisposes the HR-NBL patients to a high risk of adverse effects. The gonadotoxicity of alkylating chemotherapy is well-described, and total-body irradiation (TBI), previously used as conditioning for HSCT in some HR-NBL patients, may lead to gonadal failure (7, 8). However, surprisingly little is known about long-term gonadal function after childhood HSCT.

The poor prognosis has limited the study of late effects of HR-NBL. Thus far, no live birth after HR-NBL treatment has been reported. A recent retrospective study on HR-NBL survivors reported premature ovarian insufficiency (POI) in 75% (9/12) of the pubertal/post-pubertal girls (9). Other studies also list ovarian failure as a common late adverse effect in female survivors of HR-NBL (10, 11). Less is known about gonadal function in male HR-NBL survivors.

A short adult height is an acknowledged sequela of childhood HR-NBL (9, 12–15), typically associated with TBI. Most probably multiple mechanisms underlie the growth failure, among them also retinoic acid treatment by leading to premature closure of growth plates and thereby premature cessation of growth (16).

To elucidate the influence of childhood HSCT and HR-NBL on gonadal outcome, we investigated the pubertal development and indicators of gonadal function in long-term survivors of HR-NBL. We also systemically analyzed the childhood and pubertal growth in our cohort to better understand the growth deficit in HR-NBL.

## Materials and Methods

### Study Population

A national cohort of long-term (>10 years) survivors of HR-NBL treated at the University Hospitals in Finland between 1980 and 2000 were invited to participate in this follow-up study. Of the identified 23 long-term survivors, 20 (87%) consented and completed the current follow-up visit; laboratory examinations were obtained for 19/20 subjects. The clinical and treatment characteristics of the enrolled cohort (17), and the outcome of the patients treated at Helsinki University Hospital (4, 18) have been previously published. The characteristics of the cohort are shown in Table 1. The control group comprised 20 healthy, age- and sex-matched paired controls, as previously described (17). Informed written consent was received from all the study subjects, and in case of pediatric participants, also from the guardians. The institutional review board approved the study, and it was carried out in accordance with the Declaration of Helsinki.

**Table 1:** Clinical and treatment characteristics, and gonadal hormone concentrations [median [interquartile range] or (range)] in the survivors of high-risk neuroblastoma (HR-NBL; *n* = 20), and their healthy controls (*n* = 20).

	**HR-NBL (*n* = 20)**	**Control (*n* = 20)**	***p***
Gender (female/male)	11/9	11/9	–
Age at HR-NBL diagnosis (years) (range)	1.6 (0.2–3.6)		
Age at HSCT (years) (range)	2.3 (1.0–4.1)		
TBI (yes/no)	10/10		
Local RT	14/20		
CED (mg/m^2^) (range)	9,900 (5,600–62,400)	–	–
DIE (mg/m^2^) (range)	120 (0–210)	–	–
Cisplatin cumulative (mg/m^2^) (range)	320 (180–820)	–	–
Median follow-up time (years) (range)	19 (13–27)		
Age (years) (range)	21.7 (15.9–30.1)	22.1 (15.6–30.0)	0.92
BMI (kg/m^2^)	20.6 [19.0; 23.6]	22.3 [21.4; 26.3]	0.055
Height SDS	−3.0 [−3.4; −1.0]	0.3 [−0.7; 1.2]	*<0.001*
Sitting height (range)	0.519 (0.44–0.55)	0.532 (0.51–0.56)	*p < 0.001*
	**HR-NBL male (*n* = 9)**	**Male control (*n* = 9)**	
Testis volume (ml)	8.5 [5.8–17]	39 [29–49]	*p < 0.001*
Inhibin B (ng/l)	<10 [ <10–26] *n* = 8	166 [138–173]	*p < 0.001*
Testosterone (nmol/l)*	12.3 [8.5; 16.3] *n = 6	18.9 [14.8; 26.6]	*p = 0.05*
AMH (μg/l)	2.1 [0.30; 3.30] *n* = 8	3.4 [2.4; 11.6]	*p* = 0.07
FSH (IU/l)*	26.3 [14.2; 36.1] *n = 6	3.70 [2.2; 7.3]	*p < 0.001*
LH (IU/l)*	10.0 [8.3; 19.3] *n = 6	4.1 [3.1; 6.8]	*p = 0.005*
	**HR-NBL female (*n* = 11)**	**Female control (*n* = 11)**	
AMH (μg/l) (range)	<0.2 (<0.02–0.29)	1.7 (0.5–6.6)	*p < 0.001*
Estradiol (μmol/l) (range)*	0.1 *n* = 1	0.28 (0.11–0.65)	NA
FSH (IU/l) (range)*	7.5 *n* = 1	4.9 (1.7–8.4) *n* = 4	NA
LH (IU/l) (range)*	4.1 *n* = 1	4.3 (0.7–35.6) *n* = 4	NA

Median (range) or median [interquartile range].

HR-NBL, high-risk neuroblastoma; HSCT, hematopoietic stem cell transplantation; TBI, total-body irradiation; CED, cyclofosfamide equivalent dose; DIE, doxorubicin equivalent dose (anthracycline chemotherapy); BMI, body mass index; testo, testosterone; sitting height, sitting height to total height ratio; AMH, anti-Müllerian hormone.

* Ten of the 11 female survivors and three male survivors were on hormone (estrogen/testosterone) substitution, and 7/11 control females were on estrogen containing e-pills, and their LH, FSH, E2/testosterone values were excluded from the analysis.

*Italic font refers to significant p values*.

### Clinical and Laboratory Evaluations

**Figure 1:**
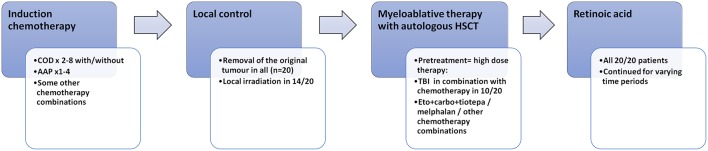
Oncologic treatment in the 20 long-term survivors of high-risk neuroblastoma. COD, cyclophosphamide + vincristine + DTIC; AAP, ciplatin + doxorubicin; HSCT, hematologic stem cell transplantation; TBI, total-body irradiation.

All subjects (HR-NBL *n* = 20; controls *n* = 20) had physical examination by the same physician (AS). Blood samples (HR-NBL *n* = 19; controls *n* = 20) for pituitary and gonadal hormone analyses were drawn after an over-night fast between 8.30 and 9.15 a.m. on the examination day. Data on pubertal development, pubertal induction and sex hormone substitution were collected from hospital records, and confirmed with a questionnaire and interview. The oncologic treatments are described in Figure 1. The cumulative dose of alkylating chemotherapy was calculated using cyclophosphamide equivalent dose (CED) as described (19). The cumulative doxorubicin, the only used anthracycline, and cisplatin doses were calculated. Testis size exceeding 15 ml was regarded normal (20). Gonadal failure was defined as: (A) absent pubertal development, (B) history of increased gonadotropins [FSH exceeding 10.4 IU/l for males (21)] after spontaneous puberty needing hormonal replacement therapy (HRT), (C) small post-pubertal testis size (<15 ml) as a predictive sign of non-functional spermatogenesis (20). Sufficient growth data with at least biannual measurement of height until 15 years of age were obtained for 17/20 HR-NBL subjects. All height measurements were transformed into standard deviation scores (SDS) according to Finnish growth standards (22). For each subject, an individual growth curve was drawn. We also analyzed height SDS at the diagnosis of HR-NBL, at the end of treatment, at pre-pubertal age (~5 years after completing the treatment but before pubertal growth), at pubertal peak height velocity, and at final height. The magnitude of pubertal growth spurt (greatest growth velocity) was evaluated by inspecting each individual growth curve.

### Statistical Analyses

Data analyses were performed using IBM SPSS statistical software for Windows, Version 25.0 (IBM Corp., Armank, NY, U.S.). Due to the small sample size, the non-parametric Mann-Whitney U test was used for all group comparisons, and data are presented as median ± interquartile range or range, as appropriate. Fischer's exact test was used to compare binary variables between the study groups and subgroups. All statistical analyses were 2-sided, and *p*-values ≤0.05 were considered significant.

## Results

### Gonadal Function

The treatment characteristics, pubertal development and indicators of gonadal function are described in Tables 2, 3. Puberty was hormonally induced due to delayed/absent pubertal development in four survivors (one male; three with TBI). None of the survivors presented with true precocious puberty. Because of early puberty (not fulfilling the criteria of precocious puberty) and/or moderate bone age advancement, four male survivors were treated with GnRH-analog (*n* = 2) or letrozole (*n* = 2) in an attempt to delay epiphyseal closure and maximize the final height (Table 2). The concentrations of gonadotropins and gonadal hormones are shown in Table 1.

**Table 2:** Pubertal development, need of hormone substitution and cumulative alkylating chemotherapy in male survivors (*n* = 9) of childhood high-risk neuroblastoma.

**Patient**	**TBI**	**Local RT^α^**	**CED (mg/m^**2**^)**	**Follow-up time (years:s)**	**Puberty initiation (spontaneous/induced)**	**Pubertal induction therapy/age**	**Testis size (ml)**	**Inhibin B (ng/l)**	**Gonadal failure with need of HRT or small testis size**	**Testosterone substitution (from age)**	**Hypothyroidism**	**Other**
**TBI TREATED**												
1	+	+	7,400	19.7	Spontaneous normally timed	No	6	11	YES; increased gonadotropins and low testosterone post-pubertally before HRT	Yes, 15 years ->	+	Bone age advancement during puberty; Letrozole^β^
2	+	–	8,650	21	Spontaneous early puberty^£^	No	9	¥N/A	YES; increased gonadotropins, decreasing testosterone before HRT	Yes, 20 years ->	+	GnRH-analog for delaying puberty
3	+	–	9,900	23	Hormonally induced	Yes/13 years	1	<10	YES, increased FSH and low testosterone before HRT	Yes, 13 years ->	+	Letrozole^β^
4	+	–	11,400	24.2	Spontaneous early^£^	No	9	<10	YES, increased gonadotropins, low testosterone and small testis size	Not currently (previously from 18 years ->)	+	GnRH-analog for delaying puberty
Summary TBI		1/4	Mean	Mean	Spontaneous puberty 3/4	Induction 1/4	Mean 6 ml	All below the reference range	4/4	4/4 (currently 3/4)	4/4	
**NO TBI**												
5	–	+	10,840	14.2	Spontaneous normally timed	No	*19*	<10	NO; high FSH but normal testosterone and testis size	No	–	BA advancement during puberty
6	–	–	46,830	13.9	Spontaneous early (no hormonal treatment)	No	*16*	26	NO; high FSH but normal testosterone and testis size	No	–	BA advancement during puberty
7	–	+	5,600	26	Spontaneous puberty	No	6	<10	YES; high FSH, low testosterone and small testis size	No	–	–
8	–	+	45,000	16.8	Spontaneous normal	No	8	<10	YES; high current FSH, normal testosterone but small testis size	No	–	–
9	–	–	5,600	27.4	Spontaneous normally-timed	No	*20*	26	NO; high current FSH and low testosterone but normal testis size	No	–	Has offspring
Summary no TBI		3/5	Mean	Mean	All with spontaneous puberty	Induction 0/5	Mean 14 ml	All below the reference range	2/5	0/4	0/4	

TBI, total-body irradiation; RT, radiotherapy; CED, cyclophosphamide equivalent dose (= cumulative dose of alkylating chemotherapy); testo, testosterone; HRT, hormone replacement therapy.

α Local RT retroperitoneal.

£ In two males, early spontaneous puberty was treated with GnRH-analog in order to suppress gonadotropins and optimize the growth potential.

β In two males, letrozole was used in order to optimize the growth potential in height by suppressing bone age advancement.

¥ In one male subject, laboratory test were not taken at current evaluation, although he had consented to participate in the study.

*Normal testis volume >15 ml marked with italic*.

**Table 3:** Pubertal development, need of hormone substitution and cumulative alkylating chemotherapy in female survivors (*n* = 11) of childhood high-risk neuroblastoma.

**Patient**	**TBI**	**Local RT^α^**	**CED (mg/m^**2**^)**	**Puberty (spontaneous or induced)/age**	**Spontaneous menarche/age**	**Ovarian failure with high gonadotropins and need of HRT**	**Estrogen substitution (±), since age**	**Current HRT**	**AMH (μg/l)**	**Hypothyroidism**	**Other**
**TBI TREATMENT**											
1	+	+	8,600	Spontaneous puberty	YES, at 13 years	YES; develops after spontaneous menarche	YES from 13 years	YES	≤0.02	–	
2	+	(+)	11,400	Spontaneous puberty	NO	YES; develops after puberty	YES, from 14 years	YES	≤0.02	+	
3	+	–	7,500	Spontaneous puberty	Yes, at 12.5 years	YES; develops after spontaneous menarche	YES, from 14 years	YES	≤0.02	–	
4	+	+	9,900	Hormonally induced at 13 years	No	YES	YES, from 13 years	YES	≤0.02	+	
5	+	+	9,900	Spontaneous puberty	YES, at 12 years	YES; develops after spontaneous menarche	YES, from 16 years	YES	≤0.02	–	
6	+	+	40,650^β^	Hormonally induced at 11.5 years	NO	YES	YES, from 11.5 years	YES	≤0.02	+	
TBI summary		5/6	14,700	Spontaneous 4/6	Menarche 3/6	6/6	6/6	6/6	All with ≤0.02	3/6	
**NO TBI TREATMENT**											
7	–	(+)	39,900^β^	Hormonally induced at 13 years	No	YES	YES, from 13 years	YES	≤0.02	+	
8	–	+	62,400^β^	Spontaneous puberty	YES, at 13.5 years	YES; develops after spontaneous menarche	YES, from 14 years onwards	YES	≤0.02	–	
9	–	(+)	9,600	Spontaneous puberty	YES at 13 years	NO	NO (but E-pills for pregnancy control)	NO	≤0.02	–	
10	–	+	5,600	Spontaneous puberty	YES, at 13.5 years	NO	NO	NO	0.40	+	History of pregnancy
11	–	+	5,600	Spontaneous puberty	YES, at 12 years	Partial; develops after spontaneous pregnancies	YES; E-pills (oligomenorrhea)	YES	≤0.02	–	History of pregnancy
No TBI summary		5/5	24,600	1/6	4/5	2/5	3/5	3/5	≤0.02 in 4/5	2/5	

α Local RT at abdominal or lumbosacral area +, other areas (+).

β Tandem HSCT, first with melphalan and the other with thiotepa ± TBI.

*CED, cyclophosphamide equivalent dose (= cumulative dose of alkylating chemotherapy); TBI, total-body irradiation; HRT, hormone replacement therapy; AMH, anti-Müllerian hormone*.

### Male Gonadal Function

Altogether 6 male survivors were defined as having gonadal failure (Table 2). In four of these, gonadal failure manifested at adolescence as small atrophic testes together with high FSH and low testosterone levels and in one as absent pubertal development. At the current examination, the male survivor group had smaller testicular volume, lower inhibin B and higher FSH (those without testosterone substitution) concentrations compared with male control group, and a trend toward lower AMH concentrations (Table 1). Three male survivors were on testosterone substitution; all the remaining male survivors presented with abnormally high FSH concentrations [>10.4 IU/l (19)] (Table 2).

Altogether 3 male survivors had testis volume >15 ml, none of them treated with TBI. Their median CED (10,800 vs. 9,300 mg/m^2^, *p* = 0.90), cumulative cisplatin (360 vs. 280 mg/m^2^, *p* = 0.56) or doxorubicin doses (0 vs. 120 mg/m^2^, *p* = 0.18) did not differ from the other survivor males. These survivor males had higher median inhibin B and AMH compared with survivor males with small testicle size (26 vs. <10 ng/ml, *p* = 0.003 and 3.4 vs. 1.1 μg/ml, *p* = 0.024, respectively). Inhibin B was below the normal reference range in all survivor males (Table 1), with no significant difference between TBI-treated and other survivor males (median <10 vs. <10 ng/ml, *p* = 0.79). There was a trend toward lower AMH levels in the TBI-treated compared with other survivor males (0.02 vs. 2.8 mg/l, *p* = 0.14).

The only male survivor with off-spring had normal testis volume but high FSH and low inhibin B level. His HR-NBL treatment had included induction chemotherapy with cisplatin and etoposide, and melphalan as the high-dose therapy, summing up with CED of 5,600 mg/m^2^. His sperm analysis showed oligo-asthenozoo-spermia with total sperm count 0.80 E^6^/ml (reference range of the laboratory >20 E^6^/ml). Sperm analysis was not performed in other male survivors.

### Female Gonadal Function

Altogether eight (73%) of the 11 female survivors were defined as having gonadal failure (TBI treated 6/6 vs. non-TBI treated 2/5) (Table 3). Among them, both two non-TBI treated females had received tandem HSCT, and the other also RT at pelvic/abdominal area (Patients 7–8, Table 3). Altogether 9/11 female survivors were currently on estrogen substitution that had been started at pubertal induction (*n* = 3), during/after puberty when gonadotropin levels continuously increased exceeding the reference range (*n* = 5) or when secondary oligo-amenorrhea manifested (*n* = 1). All the survivor females with previously diagnosed gonadal failure had presented with high LH and/or FSH prior to estrogen substitution. Despite estrogen substitution, three female survivors had a FSH level exceeding the reference range at current examination. AMH was <0.02 μg/L, the lower limit of the analysis, in all survivor females except one treated without TBI (Tables 1, 3).

Eight survivor girls entered puberty spontaneously, and 7/8 of them also had spontaneous normally-timed menarche (mean 12.7 years) but 5/8 of them manifested ovarian failure with increasing gonadotropin levels post-pubertally (Table 3). Altogether three female survivors manifested signs of preserved ovarian function and two of them had been pregnant (patients 9–11, Table 3). They had been treated with single HSCT, without TBI and melphalan as the high-dose treatment, with CED:s ≤9,600 mg/m^2^. Two of them were on estrogen-containing contraceptive pills, one for birth control and one because of oligomenorrhea. They both had therefore low FSH and LH at current examination. The only female survivor without current estrogen treatment had normal FSH and LH (Table 1).

#### Pregnancies and Off-Spring

Three HR-NBL survivors (one male) had carried/fathered altogether nine pregnancies, all treated without TBI and with CED of 5,600 mg/m^2^. One pregnancy was electively terminated; the live-born children had the following gestational ages (premature/full-term, FT) and birth weights: premature week 23/610 g; FT/3.620 kg; FT/3.640 kg; FT/3.395 kg; FT/2.850 kg; FT/3.526 kg; FT/3.370 kg; FT/3.720 kg. No miscarriages were reported by the survivor females. The preterm baby was deceased during the neonatal period. All full-term born children were healthy with no birth anomalities.

### Growth

**Figure 2:**
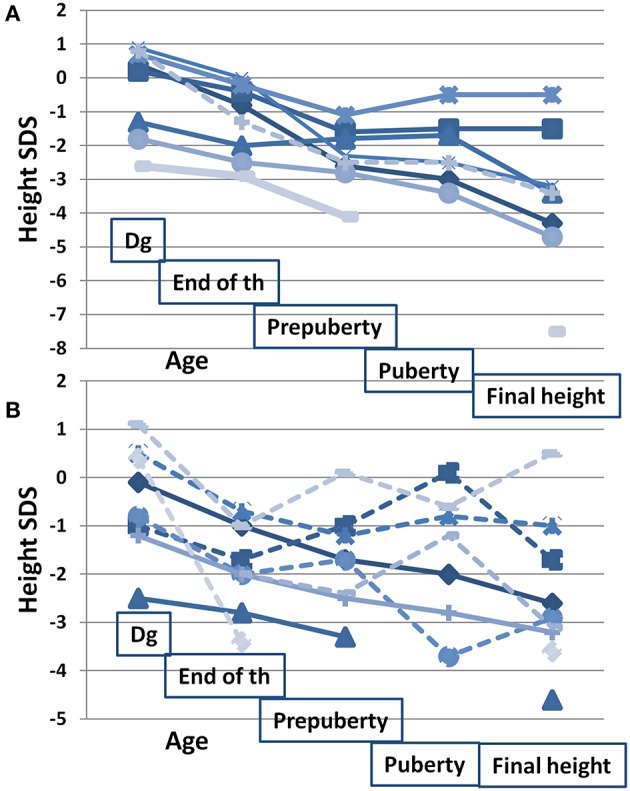
Height SDS in relation to oncologic treatment and puberty in long-term survivors of HR-NBL (*n* = 17). Height SDS measured at/before diagnosis (dg; median age at measurement 1.6 years, range 0.0–4.3), at the end of HR-NBL treatment (end of th; median 2.4 years, range 1.4–4.4), at pre-pubertal age (pre-puberty; median 7.8 years, range 6.5–8.8), at pubertal peak height velocity (puberty; median 13.1 years, range 11.8–14.8) and at final height. Growth patterns are shown separately for those with GH treatment **(A)** and for those without GH treatment **(B)**. Among the GH treated, GH treatment was started at mean age of 7.6 years (between 4 and 11). Those treated with TBI are marked with solid line and those without TBI with dotted line. Altogether 10 had hypothyroidism (7 with GH treatment; 3 without GH treatment) substituted with thyroxine.

**Figure 3:**
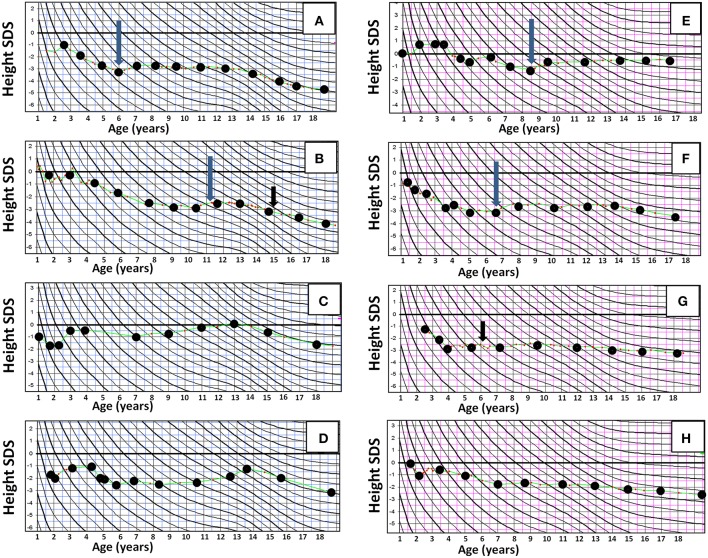
Representative individual growth curves in height in long-term survivors of high-risk neuroblastoma (HR-NBL), shown in height SD scores according to Finnish growth curves, with age in years at X axis and height SDS at Y axis. **(A–D)** Male survivors of high-risk neuroblastoma: **(A,B)** TBI treated. Growth failure begins after HR-NBL diagnosis and becomes more pronounced with lacking pubertal growth spurt. **(C)** No TBI. Normal growth velocity after HR-NBL treatment, but mildly compromised adult height due to early pubertal growth spurt. **(D)** No TBI. Growth failure after HR-NBL treatment, and early pubertal growth spurt leading to further compromised adult height. **(E–H)** Female survivors of HR-NBL. **(E)** TBI treated. Slightly compromised growth velocity after HR-NBL treatment in early childhood, but after that (with GH) normal growth and normal pubertal growth. **(F)** No TBI. Pronounced growth failure begins after HR-NBL treatment; blunted pubertal growth spurt. **(G–H)** Both TBI treated. Growth failure begins after HR-NBL treatment and is further augmented with blunted pubertal growth. Long arrow, GH treatment begins; short arrow, thyroxine substitution for hypothyroidism begins.

Figure 2 shows height SDS at different time points in relation to HR-NBL treatment and puberty (*n* = 17), separately for those with and without growth hormone (GH) therapy. The final height SDS (median −3.0 SDS, Table 1) was lower than height SDS at HR-NBL diagnosis in each subject. The pubertal growth spurt was absent or blunted in 7/17 survivors (three females, four males; 6/7 with TBI), uninterpretable in 2 females and normal in 8 survivors. Among those with pubertal growth spurt, it was precocious in three males and late in one female. At puberty, bone age was delayed in all survivor girls, while moderately (1–2 years) advanced bone maturation had been recorded in four males (2 TBI-treated). The median sitting height was lower in the survivors than the controls (Table 1). Typical individual growth curves of HR-NBL survivors with growth deficit are presented in Figure 3.

Altogether ten (50%; six female) survivors had received GH therapy, nine (90%) of them treated with TBI. Only three of them (all females) had laboratory-proven GH deficiency while others were treated based on the treating endocrinologist's judgment of the growth deficit. None of the subjects continued on GH after completion of growth. Overall the response to GH was poor. Two of the GH-treated subjects showed improved growth velocity during GH therapy. Despite the treatment, final height remained extremely low (between −4.3 and −7.5 SDS) in all the male survivors with GH therapy. They all were TBI treated, without laboratory-proven GH deficiency and 2/4 of them also had severe kidney insufficiency. Altogether 10 survivors (eight with TBI) had hypothyroidism that was timely substituted (Tables 2, 3).

## Discussion

This national cohort study explores the gonadal function and fertility after HR-NBL in detail, and it shows that most male (6/9) and female survivors (8/11) of HR-NBL present with gonadal failure at young adulthood.

All male survivors treated with TBI presented with gonadal failure as defined by increased FSH together with low testosterone concentration and small testis volume (<15 ml). All the non-TBI treated survivor males had high FSH and low inhibin B levels, although the strict definition of gonadal failure was not fulfilled in all of them. There were three non-TBI treated survivors with testicular size exceeding 15 ml, which in a previous study predicted spermatogenic recovery after childhood allogeneic HSCT (20). In our cohort, cumulative doses of alkylating agents, cisplatin or anthracyclines did not correlate with testis size, probably because treatment included also TBI in half of the patients, which confounds the correlations. Some previously reported HR-NBL survivor cohorts, treated with comparable intensive protocols, also report pubertal/gonadal failure in many survivors (9, 12, 23) but they did not investigate gonadal function in more detail, and follow-up usually did not extend to adult age. Altogether 67% of long-term survivor males presented with low inhibin B as a marker of gonadal failure in a large childhood cancer cohort (24). In that study, NBL survivors were among the most affected subgroups, our findings being in line with this.

While both inhibin B and FSH correlate with sperm cell count, they are only surrogate markers of male germ-cell function and have not succeeded in predicting azoospermia in some childhood cancer survivor cohorts (25). One study on childhood cancer survivors suggested a FSH level exceeding 10.4 IU/l to be predictive of gonadal failure (21), but another study indicated that inhibin B is a better marker of spermatogenesis than FSH or other hormones (26). Although inhibin B has not been validated as a marker of male gonadal failure after childhood cancer treatment, extremely low levels of inhibin B in our survivor males probably indicate some stage of gonadal damage and defected Sertoli cell function.

The gonadotoxicity of TBI and alkylating chemotherapy is well-known (27). In long-term survivors of allogenic HSCT, TBI was associated with smaller testicular size and lower sperm count (20). In our cohort, the only male survivor with off-spring—assumingly with best preserved germ-cell function—also presented with high FSH, low inhibin B level and low sperm count, albeit normal testicular size. His treatment did not include TBI and CED of 5,600 mg/m^2^ was among the lowest in our cohort, with melphalan as pretreatment for HSCT. Otherwise, clear associations between treatment modalities and fertility markers could not be demonstrated.

In general, Sertoli cell and germ-cell function are more vulnerable to cancer treatments than Leydig cell function (7, 27). Also many of our non-TBI treated survivor males had normal testosterone levels, while all presented with extremely low inhibin B and high FSH levels. In adult males, AMH may serve as a biomarker of subacute testicular toxicity with higher values after cisplatin and alkylating chemotherapies suggesting testicular damage or “dematuration” of Sertoli cells (28). On the contrary, our male survivors with small testicle size and several years since treatment had low serum AMH levels with raised FSH. This has been demonstrated as a typical pattern for Sertoli cell damage following childhood HSCT and predicts azoospermia (29).

POI is well-known sequela of childhood cancer (30). Exposure to irradiation and alkylating agents results in DNA damage that induces primordial follicle oocyte death, and POI (31, 32). Recent studies have provided evidence that that oocytes at all stages of development have the capacity to repair damaged DNA (33). In turn, defects in DNA repair capacity caused by cancer therapy may contribute to secondary POI in some patients due to accelerated loss of the damaged germ cells (33). Ovarian failure, as defined by either absent pubertal development or abnormally high circulating gonadotropin concentrations needing estrogen substitution, was common in our female survivors. The risk of ovarian failure was determined by TBI: all TBI-treated female survivors but only two survivors without TBI (both with tandem HSCT:s) were affected. In most cases, ovarian failure manifested after spontaneous puberty. It is notable that even those with spontaneous menarche ended up with premature menopause and HRT already few years after menarche, this observation being consistent with severely reduced ovarian reserve. AMH, a production of granulosa cells of the growing follicles (34), reflects the size of primordial follicle pool (35), and is regarded a measure of ovarian reserve (34, 36). AMH was extremely low in all but one of our female survivors, indicating reduced ovarian follicle pool. Three female survivors, all treated without TBI, had partially preserved ovarian function as indicated by pregnancies or normal gonadotropins and regular menstruation, after spontaneous puberty-menarche. Nevertheless, two of them also presented with low AMH at young adulthood, indicating increased risk of developing POI. Importantly, these women probably have preserved fertility during a narrow window, which should be considered early during their follow-up in order to provide timely information on fertility possibilities. In previous studies, the risk of POF has been highest when alkylating chemotherapy has been combined with pelvic or abdominal radiotherapy (30, 37). In our cohort, those with best preserved ovarian function were treated with melphalan as the high-dose treatment and with CED of <10,000 mg/m^2^. This finding is in line with a previous study showing better preserved ovarian function with melphalan-based conditioning compared to busulfan-containing conditioning regimens for HSCT (38).

As reported in our cohort (15) and others (9–13), growth failure is common in HR-NBL survivors. In the current study, we found that pubertal growth spurt was absent or blunted in one third of the survivors. The significance of pubertal growth on the final height was, nevertheless, rather small, since growth velocity was typically decreased already during the first years following HR-NBL treatment. It is noteworthy that many survivors had other possible causes of growth failure: hypothyroidism, GH deficiency or severe kidney dysfunction. Further mechanisms underlying growth deficit may include direct spinal growth plate damage by radiation, as suggested by the more pronounced growth failure in the TBI treated (15) and directly indicated by the lower sitting height percentage in our survivors in the current study. Still another possible cause of growth deficit is premature closure of growth plates due to retinoic acid treatment, as suggested previously (16). Indeed, some of our male survivors had advanced bone maturation in the context of normally-timed or slightly early puberty and short stature for age, which could relate to retinoic acid treatment. Three male survivors also manifested early puberty and pubertal growth spurt that seemed to compromise their final height. Further, nutritional deprivation during and after the HR-NBL treatment may contribute, explaining the slow growth rate during the childhood growth (39), as discussed before (15). As for the significance of GH deficiency, a true deficit of GH could seldom be demonstrated biochemically in our subjects. And in turn, GH treatment was unable to significantly improve the final height in most cases.

Although we describe a national HR-NBL survivor cohort, the size of each subgroup is too small for detailed analyses on the effect of each treatment modality on gonadal function. Unfortunately, sperm analysis was not performed in this study. Although inhibin B is regarded a marker of Sertoli cell function and related to germ-cell function, it does not directly measure germ-cell viability. Transvaginal ovarian ultrasound was not performed, and therefore only indirect measures of ovarian follicle pool could be used. TBI is no longer used in HR-NBL patients, but half of the cohort patients were treated without TBI, similar to HR-NBL patients today. Moreover, knowledge of the combined effects of TBI and chemotherapy is relevant for the long-term survivor women in whom fertility options are currently evaluated. The strength of the study is the long follow-up from diagnosis until young adulthood, detailed description of pubertal development and careful clinical examination.

In conclusion, we demonstrate that gonadal damage is common in long-term survivors of HR-NBL treated with HSCT. Importantly, those treated without TBI were also affected. Fertility may remain preserved in some survivors, especially in those treated with chemotherapy (melphalan)-based high-dose regimens and with moderate cumulative doses of alkylating agents. Nevertheless, gonadal vulnerability differs between patients with same treatment warranting careful clinical follow-up with appropriate regular laboratory evaluations. Importantly, even in female survivors with spontaneous menarche, fertile period may be short because of reduced primordial follicle reserve. Therefore, only a narrow window maybe offered for fertility strategies, which warrants early information about pregnancy options. Fertility counseling and option for fertility preservation should be routinely given to all HR-NBL patients at diagnosis and repeated at pubertal age in all survivors.

## Data Availability

All datasets generated for this study are included in the manuscript/supplementary files.

## Author Contributions

KJ was responsible for original planning of the larger HR-NBL study project. KJ, OM, PU, and AS contributed in planning this specific study. AS examined and interviewed all the patients and controls at the current study visit. PU did the analyses and was mainly responsible for writing the article and drawing the images. KJ, OM, and AS helped in writing the article.

### Conflict of Interest Statement

The authors declare that the research was conducted in the absence of any commercial or financial relationships that could be construed as a potential conflict of interest.
